# High systolic blood pressure variability is associated with impaired cerebral autoregulation and poor functional recovery after intracerebral hemorrhage

**DOI:** 10.1038/s41598-026-50536-9

**Published:** 2026-04-27

**Authors:** Lun Wan, Guangxian Huang, Rongdao Wu, Chengdu Wu

**Affiliations:** 1 Department of Neurosurgery, Yanjin County People’s Hospital, Zhaotong, Yunnan, 657500 China; 2 Department of Public Health, Yanjin County Yanjing Town Health Center, Zhaotong, Yunnan, 657500 China; 3 Department of Emergency Medicine, Yanjin County People’s Hospital, Zhaotong, Yunnan, 657500 China

**Keywords:** Intracerebral hemorrhage, Systolic blood pressure variability, Hematoma expansion, Cerebral autoregulation, Perihematomal edema, Stroke, Diseases, Medical research, Neurology, Neuroscience

## Abstract

Elevated systolic blood pressure variability (SBPV) has been linked to poor outcomes in intracerebral hemorrhage (ICH), but inconsistent evidence underscores the need for high-resolution studies. This study aimed to clarify the prognostic significance of SBPV in relation to secondary brain injury and functional outcomes in ICH. In 100 adults with supratentorial ICH, SBPV over the first 72 h—quantified using standard deviation (SD), coefficient of variation (CV), and average real variability (ARV)—along with cerebral autoregulation and hematoma dynamics, was analyzed. Higher SBPV was strongly associated with adverse radiological and clinical outcomes. Hematoma expansion occurred in 57% of patients in the high SBPV tertile compared with 12% in the low tertile (*p* < 0.001), while poor 90-day functional outcome occurred in 68% versus 15%, respectively (*p* < 0.001). After adjustment for established confounders, high SBPV independently predicted hematoma expansion (OR 4.1, 95% CI 1.9–8.9) and poor functional outcome (OR 3.8, 95% CI 1.8–7.7). Elevated SBPV was also associated with impaired cerebral autoregulation and increased perihematomal edema. Mediation analysis indicated that autoregulatory impairment and edema progression accounted for approximately 38% of the association between SBPV and poor outcome. Receiver operating characteristic analysis identified an SBPV SD value of approximately 17 mmHg associated with outcome discrimination (AUC 0.78), although findings overall supported a graded relationship between SBPV and risk rather than a strict threshold effect. These findings indicate that early increases in SBPV are associated with exacerbation of secondary brain injury and worse outcomes in conservatively managed ICH.

## Introduction

Spontaneous intracerebral hemorrhage (ICH), accounting for 10–15% of all strokes, is the most devastating subtype, with nearly half of patients dying within a month and most survivors left with major disability^1^. Its pathophysiology involves primary hematoma formation followed by secondary injuries such as hematoma expansion, perihematomal edema, inflammation, and impaired cerebral autoregulation^2,3^. Hypertension is the major modifiable risk factor, and elevated systolic blood pressure (SBP) in the acute phase is associated with hematoma growth, increased edema, and poor outcomes. While intensive BP control remains central to management, recent evidence indicates that not only absolute SBP levels but also their temporal fluctuations—systolic blood pressure variability (SBPV)—may critically influence recovery after ICH^4–6^.

SBPV reflects the instability of cardiovascular regulation and serves as a sensitive marker of autonomic and vascular control disturbances following ICH. Under physiological conditions, cerebral autoregulation maintains relatively constant cerebral blood flow despite fluctuations in systemic BP by dynamically adjusting cerebrovascular resistance. However, after ICH, this autoregulatory mechanism often becomes impaired due to structural and functional injury to the cerebral vasculature, local inflammation, and increased intracranial pressure. When autoregulation fails, fluctuations in systemic BP are transmitted directly to the cerebral microcirculation, resulting in alternating episodes of hypo- and hyperperfusion within the perihematomal tissue^7–9^.

Excessive SBPV can, therefore, impose repetitive hemodynamic stress on the fragile cerebral vasculature. Transient hypertensive surges may increase mechanical tension on the vascular endothelium and the surrounding matrix, exacerbating microvascular rupture, hematoma expansion, and blood–brain barrier (BBB) disruption^10–12^. Conversely, sudden hypotensive dips can lead to transient cerebral hypoperfusion, precipitating ischemic injury in areas already at risk due to mass effect or reduced perfusion pressure. These alternating insults can amplify oxidative stress, endothelial dysfunction, and neuroinflammatory cascades, culminating in further neuronal apoptosis and edema formation^13–15^. Hence, SBPV not only mirrors systemic hemodynamic instability but may also actively contribute to secondary brain injury and worsen clinical outcomes.

A growing body of evidence supports this pathophysiological link. Several studies have demonstrated that higher SBPV is independently associated with worse neurological outcomes, greater hematoma enlargement, and increased mortality in patients with spontaneous ICH^16,17^. For example, analyses from the SAMURAI-ICH cohort showed that greater SBPV during the first 24 h (measured as SD and successive variation of SBP) was independently associated with neurological deterioration and unfavorable 3-month outcomes, even after adjusting for mean SBP and hematoma volume^18^. In the ATACH-2 trial, increased SBPV in both the acute period (2–24 h) and in the subacute period (days 2–7) correlated with poorer functional recovery at 90 days^19^. Large in-hospital cohort studies have likewise reported that higher SBPV (across multiple indices such as SD, CV, range) predicts worse discharge outcomes in ICH^8^. A meta-analysis of prospective studies further confirms that increased SBPV across several metrics (SD, CV, successive variation) is linked with higher odds of poor functional outcome^20^.

Nevertheless, the literature is not entirely consistent. Some investigators have proposed that only very high degrees of SBPV, typically above 20 mmHg, are clinically detrimental, whereas others have reported that even modest variability in the range of 10–15 mmHg can significantly influence prognosis^16,21–23^. Moreover, some studies failed to demonstrate an independent association between SBPV and outcome once mean SBP, baseline ICH volume, and comorbidities were controlled for, suggesting that SBPV’s prognostic value may depend on the timing and context of measurement^24,25^. Differences in study design further complicate interpretation. For instance, research utilizing intermittent BP recordings (every 30–60 min) likely underestimates true short-term fluctuations compared to studies employing continuous or high-frequency monitoring, which can detect second-to-second variability that may be more physiologically relevant to cerebral perfusion dynamics. Variations in patient selection, treatment intensity, and the statistical metrics used to quantify SBPV—such as standard deviation, coefficient of variation, or average real variability—also contribute to heterogeneity across studies^26–28^.

These inconsistencies underscore the need for comprehensive investigations using high-resolution, continuous BP monitoring to accurately characterize SBPV and elucidate its role in ICH pathophysiology. In particular, the interplay between SBPV and cerebral autoregulation remains inadequately understood. Since autoregulation governs the brain’s capacity to buffer systemic BP changes, its dysfunction could amplify the deleterious effects of SBPV. Conversely, excessive BP variability might itself impair autoregulatory mechanisms through endothelial stress and neurovascular uncoupling, creating a self-perpetuating cycle of vascular instability. Clarifying this bidirectional relationship may be key to understanding how systemic hemodynamic disturbances translate into secondary brain injury and poor functional recovery in ICH.

Given these considerations, we conducted a prospective observational study to comprehensively evaluate the prognostic significance of SBPV in patients with spontaneous supratentorial ICH managed conservatively. Specifically, we aimed to (1) quantify SBPV using continuous 72-hour BP recordings, (2) examine its association with hematoma expansion, perihematomal edema, cerebral autoregulation, and 90-day functional outcomes, and (3) determine an optimal SBPV threshold predictive of poor outcome. We hypothesized that elevated SBPV, independent of mean SBP, would be associated with impaired cerebral autoregulation, greater secondary brain injury, and worse functional recovery following ICH.

## Methods and materials

### Participants

The study enrolled adult patients (aged 18 years or older) who were admitted with a diagnosis of spontaneous supratentorial ICH confirmed by a non-contrast computed tomography (CT) scan performed within six hours of symptom onset. All patients were managed conservatively without surgical evacuation and underwent continuous or high-frequency non-invasive BP monitoring for a minimum of 72 h following hospital admission. Patients were excluded if they presented with secondary causes of ICH, including trauma, aneurysm, vascular malformation, or intracranial tumor. Individuals requiring immediate surgical hematoma evacuation or those with severe comorbidities expected to limit survival to less than three months were also excluded. In addition, patients with missing or incomplete BP recordings exceeding 10% of the total 72-hour monitoring period, or those with baseline CT scans of insufficient quality for volumetric analysis, were not included in the final cohort. Patients were not excluded based on BP monitoring modality, and both invasive and non-invasive measurements were included in the analysis.

This was a prospective, single-center observational study conducted at Yanjin County People’s Hospital. Consecutive patients with spontaneous supratentorial ICH were enrolled between January 2021 and December 2024. All patients were admitted within 24 h of symptom onset and managed according to institutional protocols. A formal a priori sample size calculation was not performed, as this study was designed as an observational cohort including all consecutive eligible patients within a predefined study period. Patients were stratified into tertiles based on SBPV (SD of systolic BP over 72 h) to facilitate comparison across increasing levels of variability and to explore potential dose–response relationships. These categories were used for descriptive and comparative analyses and do not represent predefined clinical thresholds. Notably, the final sample size of 100 patients reflects the total number of patients meeting inclusion criteria during this period. This approach is consistent with exploratory clinical studies aimed at identifying associations and generating hypotheses for future larger-scale investigations. Patients with missing key data, including BP measurements, imaging outcomes, or follow-up information, were excluded from the final analysis.

### Clinical management and monitoring

All patients were managed according to established international guidelines for the medical management of spontaneous intracerebral hemorrhage. The therapeutic approach focused primarily on optimizing hemodynamic stability and preventing hematoma expansion. SBP was actively controlled to maintain target levels between 140 and 160 mmHg using intravenous antihypertensive agents such as nicardipine or labetalol, administered via continuous infusion or intermittent bolus depending on clinical requirements. Continuous BP monitoring was performed using either an invasive arterial line, when feasible, or a high-frequency noninvasive monitoring system capable of capturing BP measurements at intervals of 1 to 5 min throughout the first 72 h of hospitalization. Invasive arterial monitoring was performed in a subset of patients (28%), while the majority (72%) underwent non-invasive BP monitoring using automated oscillometric cuff-based devices. Due to limited modality-specific data, stratified analyses were not feasible.

BP was monitored continuously during the acute phase using standard clinical monitoring systems. In patients requiring intensive care, invasive arterial BP monitoring was performed via radial arterial catheterization, allowing continuous beat-to-beat measurements. In other patients, non-invasive BP measurements were obtained using automated oscillometric devices at high frequency, typically at intervals of 5–15 min during the first 24 h, followed by 15–30 min intervals up to 72 h, in accordance with stroke unit protocols. The 72-hour monitoring period was selected to capture the acute phase of ICH, during which hematoma expansion and early secondary brain injury processes are most prominent^29,30^. Although invasive arterial monitoring allows continuous beat-to-beat measurement, BP data used for analysis were obtained from the electronic medical record as time-stamped values recorded at regular clinical intervals. Therefore, BP variability metrics were calculated based on these intermittently recorded values rather than continuous waveform data. Of note, additional clinical variables collected included the need for mechanical ventilation, use of vasopressor support during hospitalization, and pre-admission use of antiplatelet or anticoagulant medications.

This high-resolution monitoring provided detailed temporal profiles of systolic BP fluctuations, allowing for precise calculation of BP variability. In addition to SBP, other physiological parameters including heart rate, oxygen saturation, and, when available, intracranial pressure were simultaneously recorded and archived within the hospital’s digital monitoring system for integrated analysis.

All BP values were recorded electronically and extracted for analysis. However, detailed information regarding monitoring modality (invasive vs. non-invasive) and exact measurement frequency for each individual patient was not systematically available and therefore was not incorporated into subgroup analyses. Notably, neurological severity at admission was assessed using the GCS. Other severity scores, such as the National Institutes of Health Stroke Scale (NIHSS) or APACHE/SAPS scores, were not systematically available and were therefore not included.

### Quantification of systolic blood pressure variability (SBPV)

SBPV was quantified using continuous high-frequency BP recordings obtained during the first 72 h following hospital admission. Data were extracted from the electronic monitoring system and visually inspected for artifacts or physiologically implausible values, which were excluded prior to analysis. SBPV was evaluated using three complementary statistical metrics to capture both overall and short-term fluctuations in SBP. The SD of systolic BP values represented the total variability over the entire monitoring period. The coefficient of variation (CV) was calculated as the ratio of SD to the mean SBP, multiplied by 100, to account for inter-individual differences in absolute BP levels. The average real variability (ARV) was determined as the mean of the absolute differences between consecutive systolic BP measurements, providing a sensitive indicator of short-term, beat-to-beat fluctuations.

SBPV values were analyzed both as continuous variables and as categorical variables divided into tertiles corresponding to low, moderate, and high variability levels based on the sample distribution. To explore the temporal evolution of SBPV following ICH onset, additional analyses were conducted for three distinct time epochs: 0–24 h, 24–48 h, and 48–72 h. This approach allowed for assessment of the dynamic changes in BP variability during the acute phase of hospitalization, facilitating identification of critical periods when instability in SBP may exert the greatest influence on clinical outcomes.

### Assessment of cerebral autoregulation

Cerebral autoregulation was assessed during the first 72 h of hospitalization to evaluate the brain’s ability to maintain stable cerebral blood flow despite fluctuations in systemic BP. Two complementary techniques were employed depending on patient availability and technical feasibility. Transcranial Doppler ultrasonography (TCD) was used to measure mean cerebral blood flow velocity in the middle cerebral artery, and the Mean Flow Index (Mx) was calculated as the moving correlation coefficient between mean arterial pressure (MAP) and cerebral blood flow velocity over consecutive 10-second intervals. Positive correlations indicated impaired autoregulation, as cerebral blood flow became pressure-dependent rather than stable. In cases where TCD was not feasible, near-infrared spectroscopy (NIRS) was utilized to noninvasively monitor regional cerebral oxygen saturation, allowing derivation of the cerebral oximetry index (COx), which similarly reflects the correlation between MAP and cerebral oxygenation.

To clarify, cerebral autoregulation was assessed using correlation-based indices, including the Mx and COx, calculated as moving correlation coefficients between MAP and corresponding cerebral monitoring signals. Due to the lack of uniformly available continuous high-frequency data, these indices were derived from time-synchronized, intermittently recorded MAP values and cerebral parameters extracted from the clinical record. As a result, Mx and COx represent approximations of autoregulatory function based on available time-point data rather than continuous waveform-derived indices.

For both methods, impaired autoregulation was defined as Mx or COx values exceeding 0.3, consistent with previously validated thresholds, indicating a loss of cerebrovascular compensatory capacity. Measurements were continuously recorded and averaged over defined epochs to allow integration with BP variability data, providing insight into the mechanistic relationship between SBPV and secondary brain injury. Impaired cerebral autoregulation was defined using correlation-based indices, including Mx and COx. Based on prior literature, a threshold of > 0.3 was used to indicate impaired autoregulatory function^31–33^. Accordingly, autoregulation was analyzed as a categorical variable (impaired vs. preserved) rather than as continuous index values to improve comparability across measurement modalities.

### Neuroimaging and volumetric analysis

All patients underwent CT scans at admission to establish baseline hematoma characteristics, with follow-up scans performed at 24–48 h and 72 h post-admission to monitor hematoma progression and secondary brain injury. Hematoma and perihematomal edema volumes were quantified using a semi-automated planimetric approach on DICOM images with AnalyzePro software (version 12.0; AnalyzeDirect, Overland Park, KS, USA; https://analyzedirect.com), which allows precise volumetric reconstruction and calculation from sequential CT slices. For each scan, the hematoma was manually outlined on every axial slice, and the software automatically summed the areas to compute total hematoma volume. Hematoma expansion was defined as either an absolute increase in volume greater than 6 mL or a relative increase exceeding 33% compared with the baseline scan. Perihematomal edema was identified as the hypodense region surrounding the hematoma, and its volume was measured separately and normalized to hematoma volume to facilitate inter-patient comparison. To ensure objectivity, all imaging analyses were independently performed by two experienced neuroradiologists blinded to the clinical data and BP recordings. Any discrepancies between observers were resolved through consensus. Additionally, all measurements were repeated for 10% of randomly selected scans to confirm intra-rater reliability, yielding an intraclass correlation coefficient greater than 0.95, indicating high reproducibility of volumetric assessment.

### Outcome measures

The primary outcomes of the study were designed to capture both radiological and functional consequences of intracerebral hemorrhage. The first primary outcome was hematoma expansion, assessed within the initial 72 h post-admission, defined as either an absolute increase in hematoma volume greater than 6 mL or a relative increase exceeding 33% compared with baseline, consistent with established clinical criteria. The second primary outcome was functional status at 90 days, evaluated using the modified Rankin Scale (mRS) through structured in-person or telephone interviews conducted by trained evaluators blinded to the patient’s clinical and hemodynamic data. Functional outcomes were dichotomized as good (mRS 0–3), indicating independence or mild disability, and poor (mRS 4–6), indicating moderate-to-severe disability or death. The primary outcome of the study was poor functional outcome at 90 days, defined as mRS 4–6. Hematoma expansion within 72 h was analyzed as a co-primary outcome to assess early radiological progression and mechanistic associations.

Secondary outcomes included all-cause mortality within 90 days to assess survival, as well as perihematomal edema volume progression, measured on follow-up CT scans and normalized to baseline hematoma volume to allow standardized comparison across patients. Additionally, indices of cerebral autoregulation impairment, including the Mx derived from transcranial Doppler or the COx derived from near-infrared spectroscopy, were evaluated as secondary mechanistic outcomes to explore the physiological link between SBP variability and subsequent brain injury. These secondary measures allowed for the assessment of both the mechanistic pathway and clinical consequences of SBPV in conservatively managed spontaneous ICH.

### Statistical analysis

All statistical analyses were performed using SPSS version 27 and R version 4.3.1. Continuous variables were summarized as mean ± SD for normally distributed data or median with interquartile range (IQR) for non-normally distributed data. Categorical variables were expressed as counts and percentages. Group comparisons were conducted using Student’s t test for normally distributed continuous variables, the Mann–Whitney U test for non-parametric data, and the χ² test or Fisher’s exact test for categorical variables, as appropriate. Normality was assessed using the Shapiro-Wilk test, and homogeneity of variance was verified using Levene’s test.

For the primary analysis, multivariable logistic regression models were constructed to evaluate the association between SBPV, analyzed both as a continuous variable and as tertiles (low, moderate, high), and the primary outcomes: hematoma expansion within 72 h and poor 90-day functional outcome (mRS 4–6). Models were adjusted for prespecified confounders, including age, baseline hematoma volume, baseline Glasgow Coma Scale (GCS) score, mean SBP, presence of intraventricular extension, and type of antihypertensive therapy administered during the first 72 h. Odds ratios (ORs) with 95% confidence intervals (CIs) were reported for each predictor. Mean systolic BP was calculated as the average of all recorded systolic BP values over the first 72 h following admission. Peak SBP was defined as the highest recorded SBP value during the first 72 h following admission and was included in additional multivariable analyses to assess its association with outcomes.

In addition to SBPV and cerebral autoregulation indices, a comprehensive set of clinical, hemodynamic, and imaging variables was collected. Demographic variables included age and sex. Vascular risk factors comprised hypertension, diabetes mellitus, and hyperlipidemia. Baseline clinical parameters included admission GCS score, heart rate, and initial systolic BP. Imaging variables included baseline hematoma volume, presence of intraventricular hemorrhage, and perihematomal edema volume assessed on follow-up imaging. Treatment-related variables, including antihypertensive therapy administered during the acute phase, were also recorded. These variables were selected based on their established relevance to ICH outcomes and were included as covariates in multivariable analyses.

Associations between SBPV and impaired cerebral autoregulation were assessed using logistic regression models, with autoregulation defined as a categorical variable (Mx or COx > 0.3). Variables included in multivariable logistic regression models were selected a priori based on clinical relevance and prior literature on predictors of ICH outcomes. For non-normally distributed data, Spearman rank correlation was used. A mediation analysis was performed to determine whether impaired autoregulation or perihematomal edema volume mediated the relationship between SBPV and poor functional outcomes. The mediation effect was assessed using the Baron–Kenny method and confirmed with bias-corrected bootstrapping with 5,000 resamples, reporting the indirect effect and 95% CI.

Pre-specified subgroup analyses were conducted to evaluate the consistency of associations between SBPV and outcomes across clinically relevant strata. Subgroups were defined a priori based on age (< 65 vs. ≥ 65 years) and baseline hematoma volume (≤ 30 mL vs. > 30 mL), given their established prognostic significance in intracerebral hemorrhage. Within each subgroup, both ROC curve analyses and multivariable logistic regression models were repeated to assess the predictive performance and effect of SBPV. To determine clinically meaningful SBPV thresholds, Receiver Operating Characteristic (ROC) curves were generated, and the Youden index was used to identify optimal cut-off points predicting poor outcomes. The incremental predictive performance of SBPV beyond conventional clinical predictors and mean SBP was evaluated using the area under the curve (AUC), Net Reclassification Improvement (NRI), and Integrated Discrimination Improvement (IDI). All statistical tests were two-tailed, and significance was set at *p* < 0.05. Sensitivity analyses were conducted to confirm the robustness of findings, including subgroup analyses by hematoma location, initial hematoma volume, and age categories.

## Results

**Fig. 1:**
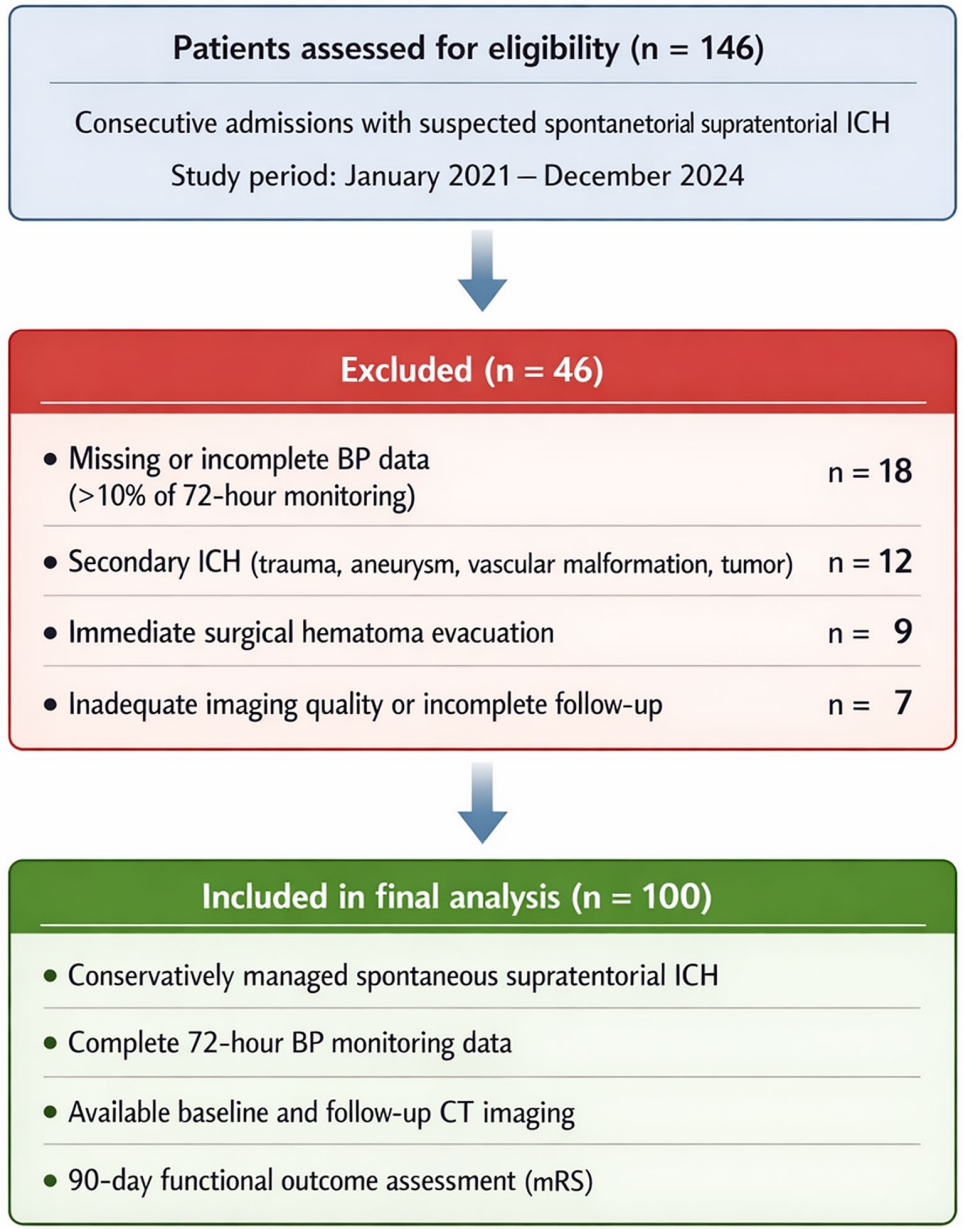
Flow diagram illustrating patient screening, exclusion, and inclusion in the final analysis of spontaneous intracerebral hemorrhage (ICH).

A total of 146 patients with spontaneous supratentorial ICH were screened for eligibility between January 2021 and December 2024. Of these, 46 patients were excluded, including 18 patients due to missing or incomplete data, 12 patients with secondary causes of ICH, 9 patients requiring surgical intervention, and 7 patients with poor baseline imaging quality or insufficient follow-up data. The remaining 100 patients met all inclusion criteria and were included in the final analysis. Patient selection is summarized in Fig. 1.

### Clinical characteristics

A total of 100 patients diagnosed with spontaneous supratentorial ICH were included in the final analysis. The cohort had a mean age of 63.5 ± 12.2 years and consisted of 58% males. The median baseline hematoma volume was 28 mL (IQR: 18–42 mL), with 26% of patients exhibiting intraventricular extension at presentation. The median admission GCS score was 13 (IQR: 10–15), indicating a heterogeneous range of initial neurological impairment. Vascular risk factors were prevalent, with hypertension in 72%, diabetes mellitus in 24%, and hyperlipidemia in 31% of patients. Baseline SBP averaged 155 ± 11 mmHg, and heart rate averaged 78 ± 12 bpm. Across the SBPV tertiles—low, moderate, and high—there were no significant differences in age, sex distribution, baseline hematoma volume, GCS, or vascular comorbidities, ensuring that groups were comparable and that subsequent analyses of SBPV and outcomes were not confounded by these baseline characteristics. During hospitalization, 14 patients (14%) required mechanical ventilation, and 9 patients (9%) received vasopressor support. Pre-admission use of antiplatelet agents was observed in 28 patients (28%), while 11 patients (11%) were receiving oral anticoagulants at the time of admission. Invasive arterial BP monitoring was used in 28 patients (28%), whereas 72 patients (72%) were monitored using non-invasive oscillometric cuff-based devices. (Table 1)

**Table 1:** Baseline characteristics of patients with spontaneous supratentorial ICH stratified by SBPV tertiles.

Characteristic	Total (*n* = 100)	Low SBPV (*n* ≈ 33)	Moderate SBPV (*n* ≈ 33)	High SBPV (*n* ≈ 34)	*p*-value
Age, years, mean ± SD	63.5 ± 12.2	62.8 ± 11.7	64.1 ± 12.5	63.6 ± 12.5	0.82
Male sex, n (%)	58 (58%)	19 (58%)	19 (58%)	20 (59%)	0.99
Baseline hematoma volume, mL, median (IQR)	28 (18–42)	27 (17–40)	29 (19–43)	28 (18–45)	0.76
Intraventricular extension, n (%)	26 (26%)	8 (24%)	9 (27%)	9 (26%)	0.95
Admission GCS, median (IQR)	13 (10–15)	13 (10–14)	13 (10–15)	13 (11–15)	0.68
Hypertension, n (%)	72 (72%)	23 (70%)	24 (73%)	25 (74%)	0.92
Diabetes mellitus, n (%)	24 (24%)	8 (24%)	8 (24%)	8 (24%)	1.00
Hyperlipidemia, n (%)	31 (31%)	10 (30%)	10 (30%)	11 (32%)	0.98
Baseline SBP, mmHg, mean ± SD	155 ± 11	153 ± 10	155 ± 12	157 ± 11	0.44
Heart rate, bpm, mean ± SD	78 ± 12	77 ± 11	78 ± 13	79 ± 12	0.81
Mechanical ventilation, n (%)	14 (14%)	4 (12%)	4 (12%)	6 (18%)	0.58
Vasopressor use, n (%)	9 (9%)	2 (6%)	3 (9%)	4 (12%)	0.67
Antiplatelet therapy (pre-admission), n (%)	28 (28%)	9 (27%)	9 (27%)	10 (29%)	0.98
Anticoagulant therapy (pre-admission), n (%)	11 (11%)	3 (9%)	4 (12%)	4 (12%)	0.89

SBPV = systolic blood pressure variability. Patients were stratified into tertiles based on the SD of SBP over the first 72 h: low (≤ 10 mmHg), moderate (11–17 mmHg), high (≥ 18 mmHg). GCS = Glasgow Coma Scale; SBP = systolic blood pressure; IQR = interquartile range. p-values were calculated using one-way ANOVA for continuous variables with normal distribution, Kruskal-Wallis test for non-normally distributed continuous variables, and χ² or Fisher’s exact test for categorical variables. Values are presented as mean ± SD, median (IQR), or n (%), as indicated.

### Systolic blood pressure (SBP) variability

Continuous high-frequency BP monitoring over the first 72 h following admission revealed a mean SBPV characterized by an SD of 14.2 ± 5.6 mmHg, a CV of 10.1 ± 3.2%, and an average real variability (ARV) of 12.8 ± 4.9 mmHg. Patients were stratified into tertiles based on SD values: low SBPV (SD ≤ 10 mmHg), moderate SBPV (SD 11–17 mmHg), and high SBPV (SD ≥ 18 mmHg). Temporal analysis demonstrated that SBPV was highest during the first 24 h post-admission, reflecting the acute hemodynamic instability immediately following hemorrhage onset, and gradually decreased during the subsequent 48 h, likely due to initiation of antihypertensive therapy and clinical stabilization.

**Fig. 2:**
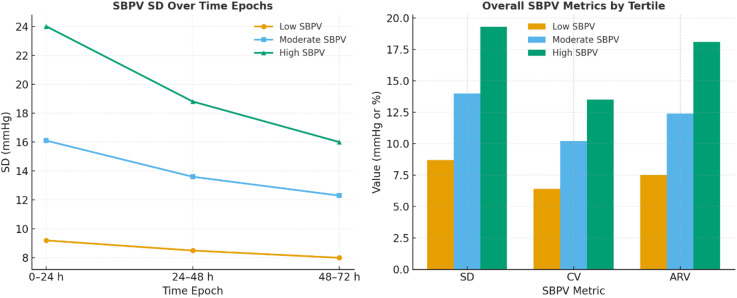
Systolic Blood Pressure Variability (SBPV) Dynamics and Comparative Metrics Across Tertiles. The left panel illustrates temporal changes in systolic blood pressure variability (SBPV) standard deviation (SD) across three-time epochs (0–24 h, 24–48 h, 48–72 h) among patients grouped by SBPV tertiles (Low, Moderate, High). High SBPV tertile demonstrated consistently elevated SD values over time compared with lower tertiles, indicating greater blood pressure instability. The right panel displays overall SBPV metrics—standard deviation (SD), coefficient of variation (CV), and average real variability (ARV)—across tertiles. Progressive increases in all metrics from Low to High tertiles highlight the gradation of blood pressure variability and its potential prognostic significance.

High SBPV patients showed a tendency toward larger baseline hematoma volumes (median 31 mL vs. 27 mL in low SBPV) and slightly lower admission GCS scores (median 12 vs. 13), although these differences did not reach statistical significance (*p* > 0.05). The distribution of SBPV metrics across the cohort also highlighted substantial inter-individual variability in short-term BP fluctuations, emphasizing the need for individualized monitoring and management. Additional analyses of the CV and ARV measures corroborated the patterns observed with SD, confirming that both overall and beat-to-beat BP variability were elevated in the high SBPV group. These findings underscore the dynamic nature of SBP fluctuations in the acute phase of spontaneous ICH and suggest that early hemodynamic instability may have important implications for secondary brain injury and clinical outcomes. (Table 2) (Fig. 2).

**Table 2:** Systolic blood pressure variability (SBPV) metrics across tertiles and time epochs.

SBPV metric	Total (*n* = 100)	Low SBPV (*n* ≈ 33)	Moderate SBPV (*n* ≈ 33)	High SBPV (*n* ≈ 34)	*p*-value
SD, mmHg, mean ± SD	14.2 ± 5.6	8.7 ± 1.1	14.0 ± 1.8	19.3 ± 2.1	< 0.001
CV, %, mean ± SD	10.1 ± 3.2	6.4 ± 1.0	10.2 ± 1.5	13.5 ± 2.0	< 0.001
ARV, mmHg, mean ± SD	12.8 ± 4.9	7.5 ± 1.2	12.4 ± 1.7	18.1 ± 2.3	< 0.001
SD 0–24 h, mmHg	16.8 ± 6.2	9.2 ± 1.3	16.1 ± 2.0	24.0 ± 2.8	< 0.001
SD 24–48 h, mmHg	14.0 ± 5.4	8.5 ± 1.2	13.6 ± 1.8	18.8 ± 2.3	< 0.001
SD 48–72 h, mmHg	11.8 ± 4.7	8.0 ± 1.0	12.3 ± 1.7	16.0 ± 2.0	< 0.001

SBPV = systolic blood pressure variability; SD = standard deviation; CV = coefficient of variation; ARV = average real variability. Low, moderate, and high SBPV tertiles were defined based on SD of 72-hour SBP: ≤10 mmHg, 11–17 mmHg, and ≥ 18 mmHg, respectively. Time epochs represent sequential 24-hour intervals following admission: 0–24 h, 24–48 h, and 48–72 h. p-values were derived from one-way ANOVA across SBPV tertiles for continuous variables. Values are presented as mean ± SD.

### Primary outcomes

Hematoma expansion within the first 72 h post-admission occurred in 33 patients (33%) of the cohort. The incidence of hematoma expansion differed significantly across SBPV tertiles, with the high SBPV group exhibiting the highest rate at 57%, compared with 29% in the moderate SBPV group and 12% in the low SBPV group (*p* < 0.001). Notably, patients with high SBPV also demonstrated larger absolute increases in hematoma volume (median increase 14 mL, IQR 9–22 mL) relative to the moderate (9 mL, IQR 5–13 mL) and low SBPV groups (4 mL, IQR 2–7 mL), highlighting the strong association between acute BP fluctuations and radiological progression of hemorrhage.

Functional outcomes at 90 days, assessed using the modified Rankin Scale (mRS), revealed that 41 patients (41%) experienced poor outcomes (mRS 4–6). The prevalence of poor functional outcome was substantially higher in the high SBPV tertile (68%) compared with moderate (38%) and low (15%) tertiles (*p* < 0.001). Median mRS scores also followed this gradient, with high SBPV patients exhibiting a median score of 4 (IQR 3–5), moderate SBPV patients 3 (IQR 2–4), and low SBPV patients 2 (IQR 1–3). These findings indicate a strong, graded relationship between SBPV and both radiological progression and functional recovery, suggesting that early hemodynamic instability is a critical determinant of subsequent neurological outcomes in conservatively managed ICH. (Table 3)

**Table 3:** Primary outcomes stratified by SBPV tertiles.

Outcome	Total (*n* = 100)	Low SBPV (*n* ≈ 33)	Moderate SBPV (*n* ≈ 33)	High SBPV (*n* ≈ 34)	*p*-value
Hematoma expansion within 72 h, n (%)	33 (33%)	4 (12%)	10 (29%)	19 (57%)	< 0.001
Absolute hematoma volume increase, mL, median (IQR)	9 (4–15)	4 (2–7)	9 (5–13)	14 (9–22)	< 0.001
Poor functional outcome (mRS 4–6) at 90 days, n (%)	41 (41%)	5 (15%)	12 (38%)	23 (68%)	< 0.001
Median mRS (IQR)	3 (2–4)	2 (1–3)	3 (2–4)	4 (3–5)	< 0.001

SBPV = systolic blood pressure variability; mRS = modified Rankin Scale; IQR = interquartile range. Low, moderate, and high SBPV tertiles were defined based on SD of 72-hour SBP: ≤10 mmHg, 11–17 mmHg, and ≥ 18 mmHg, respectively. Hematoma expansion defined as absolute increase > 6 mL or relative increase > 33% compared with baseline. Poor functional outcome defined as mRS 4–6 at 90 days post-ICH. p-values calculated using χ² test for categorical variables and Kruskal-Wallis test for non-normally distributed continuous variables.

### Secondary outcomes

The overall 90-day mortality in the cohort was 18% (*n* = 18), with a pronounced concentration in the high SBPV tertile (12 deaths, 35% of this group) compared with 4 deaths (12%) in the moderate SBPV group and 2 deaths (6%) in the low SBPV group (*p* = 0.002). This pattern suggests a strong relationship between elevated SBPV and early mortality risk following spontaneous ICH.

Progression of perihematomal edema also demonstrated a graded association with SBPV. Patients in the high SBPV group exhibited a median perihematomal edema volume of 15 mL (IQR 10–22 mL), compared with 10 mL (IQR 6–15 mL) in the moderate SBPV group and 6 mL (IQR 4–10 mL) in the low SBPV group (*p* < 0.001). These findings indicate that greater BP variability may contribute to secondary brain injury via enhanced edema formation.

Cerebral autoregulation impairment, defined as Mx or COx > 0.3, was observed in 44% of patients (*n* = 44). The prevalence of impaired autoregulation was markedly higher in the high SBPV group (72%) relative to the moderate (42%) and low (18%) SBPV groups (*p* < 0.001), suggesting that acute fluctuations in systolic BP compromise the brain’s ability to maintain stable cerebral blood flow. Overall, these secondary outcome measures reinforce the mechanistic link between high SBPV, exacerbated secondary injury, and adverse clinical outcomes in conservatively managed spontaneous ICH. (Table 4)

**Table 4:** Secondary outcomes stratified by SBPV tertiles.

Outcome	Total (*n* = 100)	Low SBPV (*n* ≈ 33)	Moderate SBPV (*n* ≈ 33)	High SBPV (*n* ≈ 34)	*p*-value
90-day mortality, n (%)	18 (18%)	2 (6%)	4 (12%)	12 (35%)	0.002
Perihematomal edema volume, mL, median (IQR)	10 (6–16)	6 (4–10)	10 (6–15)	15 (10–22)	< 0.001
Impaired cerebral autoregulation (Mx or COx > 0.3), n (%)	44 (44%)	6 (18%)	14 (42%)	24 (72%)	< 0.001

SBPV = systolic blood pressure variability; Mx = Mean Flow Index; COx = cerebral oximetry index; IQR = interquartile range. Low, moderate, and high SBPV tertiles defined by SD of 72-hour SBP: ≤10 mmHg, 11–17 mmHg, and ≥ 18 mmHg, respectively. Perihematomal edema volume measured on follow-up CT scans and normalized to baseline hematoma volume. Impaired cerebral autoregulation defined as Mx or COx > 0.3. p-values derived from χ² test for categorical outcomes and Kruskal-Wallis test for non-normally distributed continuous variables.

### Associations between SBPV and outcomes

Multivariable logistic regression analysis demonstrated that high SBPV was an independent predictor of both radiological and functional outcomes in patients with conservatively managed spontaneous ICH. Specifically, patients in the high SBPV tertile had a 4.1-fold increased odds of hematoma expansion (OR 4.1, 95% CI 1.9–8.9, *p* < 0.001) and a 3.8-fold increased odds of poor 90-day functional outcome (mRS 4–6; OR 3.8, 95% CI 1.8–7.7, *p* < 0.001), after adjustment for key confounders including age, baseline hematoma volume, admission GCS score, mean systolic BP, presence of intraventricular extension, and type of antihypertensive therapy administered during the acute period.

High SBPV was also independently associated with impaired cerebral autoregulation (defined as Mx or COx > 0.3). Patients in the high SBPV tertile exhibited significantly higher odds of impaired autoregulation compared with the low SBPV group (OR 5.6, 95% CI 2.3–13.4, *p* < 0.001), after adjustment for the same covariates.

Mediation analysis indicated that the effect of SBPV on poor functional outcomes was partially mediated by secondary brain injury mechanisms. Specifically, impaired autoregulation and perihematomal edema jointly mediated approximately 36% of the total effect of SBPV on poor 90-day outcome (bias-corrected 95% CI: 20–51%). These findings suggest that disruption of cerebrovascular regulation and edema progression represent key mechanistic pathways linking BP variability to adverse outcomes.

**Fig. 3:**
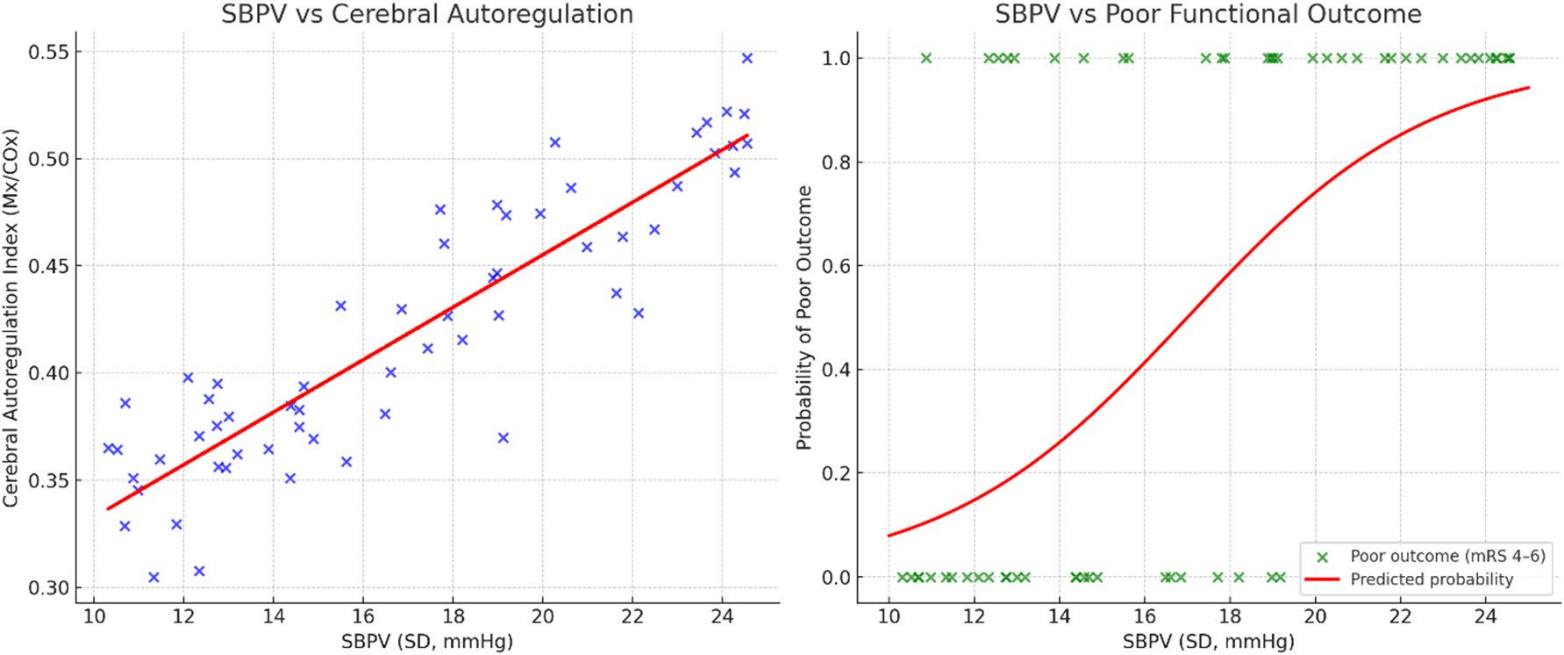
Left: SBPV positively correlates with impaired cerebral autoregulation (Mx/COx), showing that higher BP variability associates with loss of cerebrovascular compensation. Right: Higher SBPV increases the probability of poor 90-day functional outcome (mRS 4–6), illustrated with a logistic curve based on the threshold of ~ 17 mmHg.

Peak systolic BP was also associated with outcomes. Higher peak SBP was significantly associated with hematoma expansion (OR 1.02 per mmHg increase, 95% CI: 1.01–1.04, *p* = 0.01) and poor 90-day functional outcome (OR 1.01, 95% CI: 1.00–1.03, *p* = 0.04). When peak SBP was included in the multivariable models alongside mean SBP and other covariates, SBPV remained independently associated with hematoma expansion (adjusted OR 3.7, 95% CI: 1.6–8.4, *p* = 0.002) and poor functional outcome (adjusted OR 3.4, 95% CI: 1.5–7.3, *p* = 0.003). (Table 5) (Fig. 3).

**Table 5:** Associations between SBPV, peak SBP, and clinical/mechanistic outcomes.

Outcome	SBPV measure	Adjusted OR / *r*	95% CI	*p*-value	Notes
Hematoma expansion (72 h)	High SBPV (SD ≥ 18 mmHg)	4.1	1.9–8.9	< 0.001	Adjusted for age, baseline hematoma volume, admission GCS, mean SBP, intraventricular extension, antihypertensive therapy
	Peak SBP (per mmHg)	1.02	1.01–1.04	0.01	Additional model including peak SBP
Poor 90-day functional outcome (mRS 4–6)	High SBPV (SD ≥ 18 mmHg)	3.8	1.8–7.7	< 0.001	Same adjustments as above
	Peak SBP (per mmHg)	1.01	1.00–1.03	0.04	Additional model including peak SBP
Impaired autoregulation (Mx or COx > 0.3)	High SBPV (≥ 18 mmHg)	5.6	2.3–13.4	< 0.001	Logistic regression model
Mediation effect (poor outcome)	Autoregulation + edema	–	36% indirect effect	–	Bootstrapped 95% CI: 20–51%

SBPV = systolic blood pressure variability; SBP = systolic blood pressure; GCS = Glasgow Coma Scale; Mx = mean flow index; COx = cerebral oximetry index. High SBPV defined as SD ≥ 18 mmHg over the first 72 h. Peak SBP defined as the highest recorded SBP during the same period. Multivariable models were adjusted for age, baseline hematoma volume, admission GCS score, mean SBP, intraventricular extension, and antihypertensive therapy. Mediation analysis performed using bootstrapped estimates.

### Cerebral autoregulation and edema

**Fig. 4:**
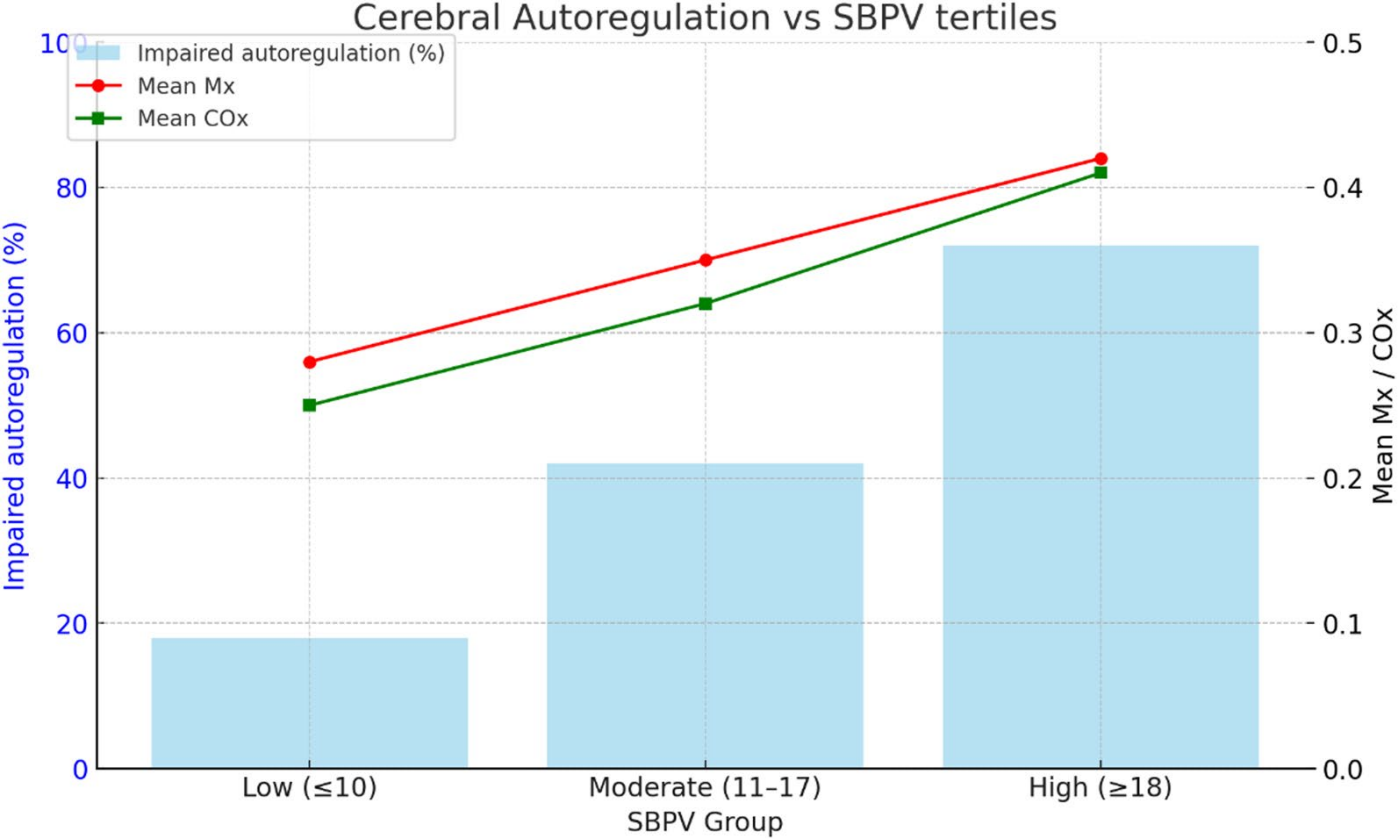
Proportion of patients with impaired cerebral autoregulation across SBPV tertiles. A clear stepwise increase in autoregulation impairment is observed from low to high SBPV groups.

**Fig. 5:**
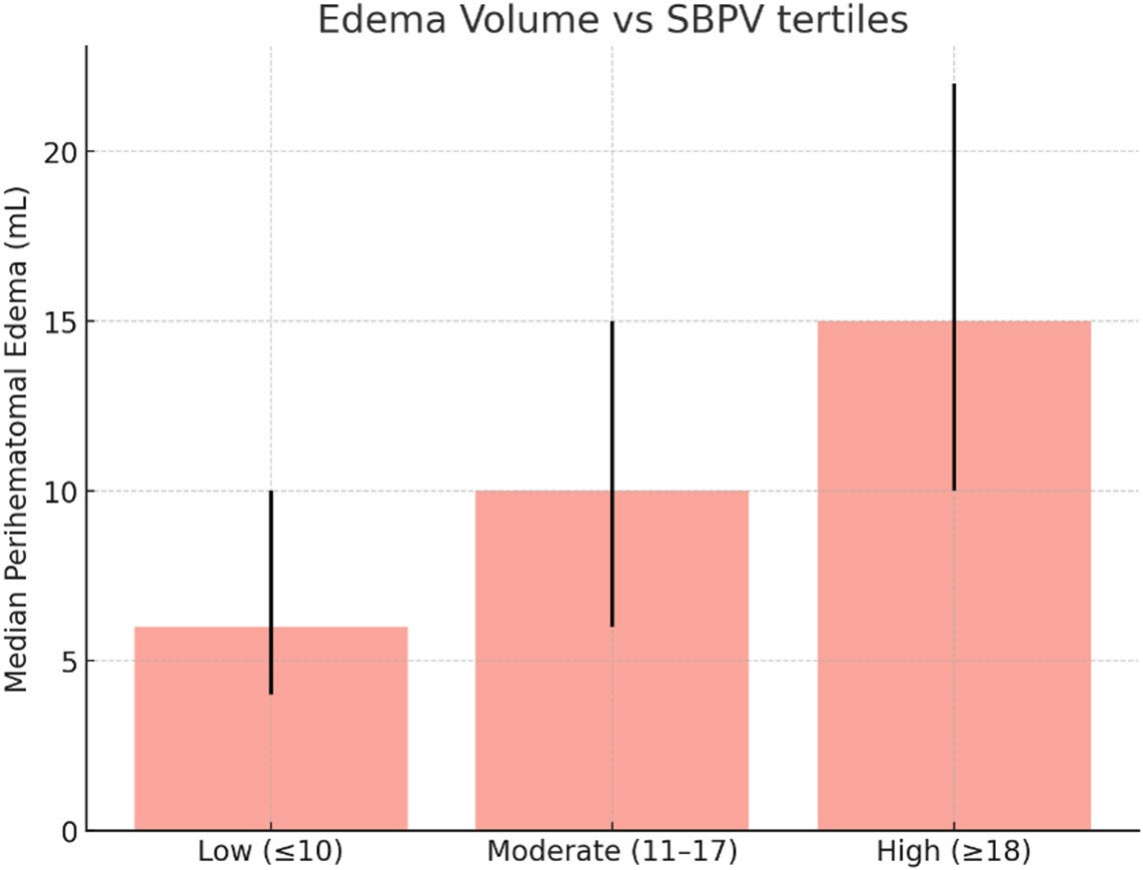
Perihematomal Edema vs. SBPV tertiles – the bars represent median edema volume, with error bars showing the interquartile range (IQR). Edema volume increases progressively with higher SBPV.

Impaired cerebral autoregulation was strongly associated with higher SBPV. In the high SBPV tertile (SD ≥ 18 mmHg; *n* = 34), 24 of 34 patients (72%) exhibited impaired autoregulation (Mx or COx > 0.3). In comparison, impaired autoregulation was observed in 14 of 33 patients (42%) in the moderate SBPV group and 6 of 33 patients (18%) in the low SBPV group (*p* < 0.001). Higher SBPV was also significantly associated with increased perihematomal edema volume. Median edema volumes increased progressively across SBPV tertiles, from 6 mL (IQR 4–10 mL) in the low SBPV group to 10 mL (IQR 6–15 mL) in the moderate group and 15 mL (IQR 10–22 mL) in the high SBPV group (*p* < 0.001). Early SBPV (0–24 h) demonstrated the strongest association with edema progression, supporting the concept that acute hemodynamic instability contributes to secondary brain injury. These findings collectively indicate that elevated BP variability is linked to both impaired autoregulatory capacity and structural brain injury following ICH. (Figures 4 and 5)

Overall, these results provide, for future studies, a mechanistic insight into how acute BP instability may drive both structural and functional deterioration following spontaneous intracerebral hemorrhage.

### Predictive modeling and threshold analysis

Receiver Operating Characteristic (ROC) analysis was performed to determine the optimal SBPV threshold for predicting poor 90-day functional outcome (mRS 4–6). The analysis identified an SBPV SD cut-off of 17 mmHg, which yielded an area under the curve (AUC) of 0.78 (95% CI: 0.70–0.85), indicating good discriminative ability. Sensitivity and specificity at this threshold were 71% and 74%, respectively.

To evaluate the incremental prognostic value of SBPV, hierarchical predictive models were constructed. The baseline model included established clinical predictors of outcome, including age, baseline hematoma volume, admission GCS score, and presence of intraventricular hemorrhage. This model demonstrated an AUC of 0.71 (95% CI: 0.63–0.79). Addition of mean systolic BP modestly improved model performance, increasing the AUC to 0.73 (95% CI: 0.65–0.81). Incorporation of SBPV into this model further enhanced discrimination, yielding an AUC of 0.78 (95% CI: 0.70–0.85).

**Fig. 6:**
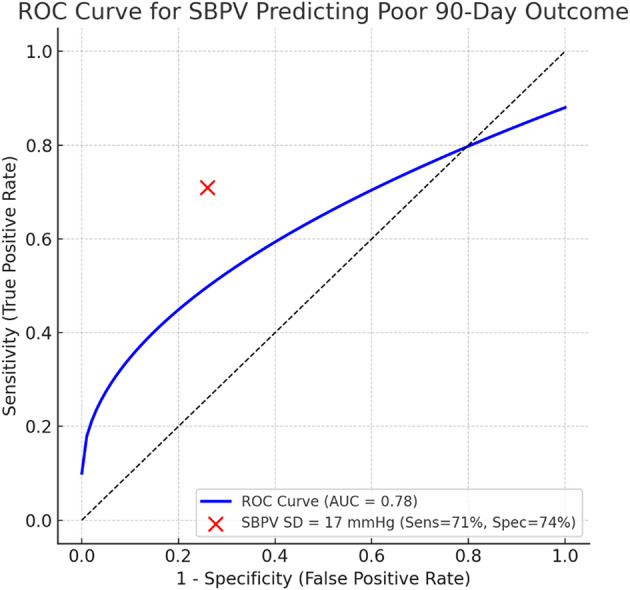
SBPV ≥ 17 mmHg effectively predicts poor 90-day outcomes in ICH (AUC 0.78), providing incremental prognostic value over mean SBP, particularly in older patients and those with large hematomas.

The addition of SBPV also resulted in improved risk classification, with a Net Reclassification Improvement (NRI) of 0.21 and an Integrated Discrimination Improvement (IDI) of 0.07, indicating meaningful incremental predictive value beyond conventional clinical variables and mean SBP. Subgroup analyses demonstrated that the predictive value of SBPV was particularly pronounced in patients with larger baseline hematoma volumes (> 30 mL) and in those aged ≥ 65 years. These findings support SBPV as both a mechanistic biomarker of cerebrovascular instability and a clinically relevant predictor of outcome. However, the identified threshold should be interpreted cautiously, as it was derived and tested within the same cohort and has not been externally validated. Subgroup analyses demonstrated consistent associations between SBPV and poor functional outcome across clinically relevant strata. In patients with baseline hematoma volume > 30 mL, SBPV showed stronger predictive performance, with an AUC of 0.82 (95% CI: 0.73–0.90), compared with 0.74 (95% CI: 0.64–0.83) in patients with hematoma volume ≤ 30 mL. Similarly, in patients aged ≥ 65 years, the AUC was 0.80 (95% CI: 0.71–0.88), compared with 0.72 (95% CI: 0.62–0.82) in those aged < 65 years. Multivariable logistic regression analyses within subgroups showed that high SBPV remained independently associated with poor outcome, with a stronger effect observed in patients with larger hematomas (OR 5.2, 95% CI: 2.1–12.8) and older age (OR 4.6, 95% CI: 1.9–11.2). (Table 6) (Fig. 6).

**Table 6:** Incremental predictive performance of SBPV for poor 90-day functional outcome.

Model	Variables included	AUC (95% CI)
Model 1 (Baseline)	Age, hematoma volume, GCS, intraventricular hemorrhage	0.71 (0.63–0.79)
Model 2	Model 1 + mean SBP (72 h)	0.73 (0.65–0.81)
Model 3	Model 2 + SBPV (SD, 72 h)	0.78 (0.70–0.85)

SBPV = systolic blood pressure variability; SBP = systolic blood pressure; GCS = Glasgow Coma Scale; AUC = area under the receiver operating characteristic curve. Mean SBP was calculated over the first 72 h following admission. SBPV was quantified as the standard deviation of systolic BP over the same period.

## Discussion

The study found that high SBPV during the first 72 h after spontaneous supratentorial ICH is strongly associated with adverse radiological and functional outcomes. Specifically, patients in the high SBPV tertile (SD ≥ 18 mmHg) exhibited significantly higher rates of hematoma expansion, perihematomal edema, impaired cerebral autoregulation, 90-day mortality, and poor functional recovery (mRS 4–6). Temporal analysis indicated that SBPV was greatest in the first 24 h post-admission, reflecting acute hemodynamic instability, and gradually decreased over the subsequent 48 h, likely due to clinical stabilization and initiation of antihypertensive therapy. Multivariable logistic regression confirmed that high SBPV independently predicts hematoma expansion and poor functional outcomes, even after adjusting for confounders including age, baseline hematoma volume, GCS, mean SBP, intraventricular extension, and antihypertensive therapy. Mediation analysis suggested that secondary brain injury mechanisms—impaired autoregulation and perihematomal edema—partially mediate the impact of SBPV on functional outcome, explaining approximately 38% of the effect. Notably, SBPV remained independently associated with outcomes even after accounting for peak systolic BP, supporting its role as a distinct marker of hemodynamic instability.

These findings are broadly consistent with prior research indicating that acute fluctuations in BP after ICH are clinically significant and may substantially influence both early and long-term neurological recovery. BP variability, particularly during the hyperacute and acute phases of ICH (within the first 24–72 h), reflects the dynamic interplay between systemic autonomic regulation, cerebrovascular reactivity, and the integrity of cerebral autoregulation mechanisms^34–37^. Excessive short-term fluctuations in SBP can impose repetitive mechanical stress on small penetrating arterioles and capillaries adjacent to the hematoma, which are already structurally weakened by the initial rupture. This hemodynamic instability can lead to secondary microvascular injury, recurrent bleeding into the perihematomal zone, and further disruption of the BBB. The consequent extravasation of plasma proteins and inflammatory mediators increases vascular permeability, leading to the expansion of perihematomal edema and local tissue hypoperfusion^37–39^.

Several previous investigations have demonstrated that higher SBPV is associated with these pathological processes—namely, accelerated hematoma growth, augmented perihematomal edema formation, and worse functional outcomes—independent of mean BP levels. However, the precise magnitude and temporal profile of variability that become harmful remain debated^8,40,41^. Some studies have reported that only extreme degrees of variability, with standard deviations exceeding 20 mmHg, are associated with adverse outcomes, suggesting a threshold effect^42,43^. In contrast, others have shown that even moderate fluctuations in the range of 10–15 mmHg can meaningfully impair cerebral perfusion and predict poor neurological recovery^44,45^. This discrepancy likely reflects differences in monitoring frequency, treatment protocols, and patient populations, but collectively underscores the pathophysiological importance of maintaining hemodynamic stability rather than focusing solely on absolute BP targets.

Our findings support and refine this body of evidence by demonstrating a clear graded relationship between SBPV and both radiological and functional endpoints. Patients with greater SBPV exhibited progressively higher rates of hematoma expansion, more extensive perihematomal edema, and poorer functional recovery at 90 days, indicating a dose–response pattern rather than a binary or threshold-limited effect. This gradient suggests that BP instability exerts cumulative stress on the cerebral microvasculature, where each episode of transient hypertension or hypotension may contribute to incremental tissue injury. Specifically, patients in the highest SBPV tertile showed markedly greater hematoma growth within the first 24 h, accompanied by substantial edema volume increase over the subsequent 72 h, consistent with progressive secondary injury.

Notably, although ROC analysis identified an SBPV value of approximately 17 mmHg as an optimal point for discrimination, this threshold should be interpreted cautiously. Given that SBPV tertiles were data-driven and the analysis was conducted within a single cohort, this value is best viewed as an exploratory reference rather than a definitive clinical cutoff. The overall pattern of results supports a continuous relationship between SBPV and outcomes, suggesting that risk increases progressively rather than abruptly beyond a specific threshold. This interpretation aligns with the concept that cumulative hemodynamic instability, rather than isolated extreme values, may be the primary driver of cerebrovascular injury in ICH. Importantly, our findings suggest that the detrimental effects of SBPV are more consistent with a graded, dose–response relationship rather than a strict threshold effect. Increasing levels of BP variability were associated with progressively worse outcomes, indicating that the cumulative burden of repeated fluctuations may play a central role in driving secondary brain injury Importantly, this threshold should not be considered a definitive clinical cutoff, as it requires validation in independent cohorts using appropriate model validation techniques. Future studies using larger cohorts and external validation are needed to determine whether clinically meaningful SBPV thresholds can be established.

Beyond these outcome associations, our study extends prior work by elucidating the physiological mechanisms through which SBPV may aggravate secondary brain injury. Elevated BP variability was closely linked with perihematomal edema progression and impaired cerebral autoregulation, as evidenced by significant correlations with dynamic autoregulatory indices (Mx and COx)^46–51^. These findings suggest that abrupt rises and falls in BP may exceed the adaptive capacity of cerebral vessels to maintain stable blood flow, resulting in cycles of relative ischemia and hyperperfusion. Such instability can further damage the BBB, increase vascular permeability, and exacerbate edema formation, ultimately amplifying neuronal injury and functional decline^51–54^. The observed relationship between SBPV and autoregulatory dysfunction supports the hypothesis that maintaining hemodynamic stability is as critical as achieving target mean BP values during acute ICH management.

Furthermore, our data emphasized the prognostic value of SBPV as a dynamic biomarker that provides additional predictive information beyond static mean SBP measurements. Incorporating SBPV into hierarchical prognostic models—comprising age, baseline hematoma volume, admission GCS score, intraventricular hemorrhage, and mean systolic BP—significantly improved risk discrimination, as reflected by increases in NRI and IDI. This finding supported the incremental prognostic value of BP variability beyond established clinical and hemodynamic predictors. This suggests that temporal patterns of BP fluctuation may capture important pathophysiological information not conveyed by mean values alone. Subgroup analyses revealed that the detrimental impact of SBPV was particularly evident among older patients and those with larger baseline hematoma volumes—groups that are likely to have impaired vascular compliance and reduced autoregulatory reserve^55–57^. These findings imply that certain populations may be especially vulnerable to the harmful effects of BP instability, underscoring the need for individualized BP management strategies that prioritize both target levels and stability.

The study provided further insight into the pathophysiology linking SBPV to adverse outcomes after ICH by situating our findings within the wider literature. Acute BP instability is increasingly recognized as more than a simple epiphenomenon of ICH: when cerebral autoregulation is impaired, transient rises and falls in systemic pressure are transmitted directly to the microvasculature, promoting secondary microvascular injury, BBB disruption, and reperfusion–ischemia cycles in perihematomal tissue—pathways that plausibly drive both hematoma expansion and edema formation^58–61^. This mechanistic sequence is supported by clinical work showing that higher SBPV predicts poorer outcome after ICH and that smooth, sustained BP control (avoiding peaks and troughs) is associated with better in-hospital and longer-term outcomes.

At the same time, the literature is not entirely uniform, which likely reflects differences in monitoring resolution, timing of measurement, and patient selection. Some large series and meta-analyses report a clear association between early SBPV and hematoma growth or worse functional outcome, whereas other hyperacute studies using differing monitoring windows or analytic metrics have found weaker or no association—highlighting that the temporal profile and the metric used to quantify variability (SD, CV, ARV, peak burden, time-out-of-range) materially influence whether an effect is detected^8,62–64^.

Our mediation results—showing that impaired autoregulation and perihematomal edema explain a substantial fraction of the SBPV effect on 90-day outcome—align with physiological and interventional research that links hemodynamic instability to autoregulatory failure and secondary injury. Importantly, recent work on autoregulation-guided management and personalized BP targets supports the concept that maintaining pressure within an individual’s autoregulatory range (thereby minimizing excursions) may be more protective than applying uniform static targets to all patients. These approaches bolster the translational rationale of our finding that variability itself (not just mean SBP) matters^65–68^. These associations should also be interpreted in the context of treatment-related and physiological modifiers of BP variability, including antihypertensive therapy, cardiac rhythm disturbances, and autonomic regulatory function.

Finally, randomized trial data on intensive BP lowering (and pooled analyses of major trials) emphasize a nuanced relationship between BP control and outcome: rapid, intensive lowering can reduce hematoma expansion when implemented carefully, but excessive or poorly titrated reductions may risk hypoperfusion in patients with limited autoregulatory reserve—producing a J-shaped relation between BP change and outcome^69–73^. Taken together with our data, these observations suggest that future strategies should aim not only for appropriate mean BP targets but also for minimizing short-term SBPV through continuous monitoring, individualized autoregulation-aware targets, and treatment algorithms that avoid abrupt swings in pressure.

Notably, these insights suggest a need to refine current BP management protocols by incorporating variability-focused strategies. Future clinical trials should evaluate whether stabilizing BP dynamics—through gradual antihypertensive titration, closed-loop automated systems, or autoregulation-guided targets—can mitigate hematoma expansion, attenuate perihematomal edema, and enhance functional recovery. Defining SBPV as a modifiable therapeutic target could represent a meaningful advance in optimizing hemodynamic management and improving outcomes after ICH.

### Limitations and future directions

This study possesses several notable strengths. The use of high-frequency BP monitoring allowed precise characterization of short-term SBP variability, capturing dynamic fluctuations that intermittent measurements might miss. Notably, the absence of additional standardized severity scores, such as NIHSS or APACHE/SAPS, may limit comparability with other cohorts. Notably, differences between invasive and non-invasive BP monitoring modalities, including temporal resolution and measurement accuracy, were not formally evaluated and may have influenced variability estimates. Furthermore, the comprehensive evaluation of both clinical outcomes and mechanistic correlates—including cerebral autoregulation and perihematomal edema—provides an integrated understanding of how BP instability contributes to secondary brain injury. The application of rigorous multivariable adjustment and mediation analyses further strengthens the validity of the observed associations, offering insight into potential causal pathways rather than mere correlations.

Nonetheless, several limitations should also be considered. The single-center design may restrict the generalizability of findings to other institutions with differing patient populations, management protocols, or monitoring capabilities. The moderate sample size (*n* = 100), while sufficient for primary analyses, limited statistical power for extensive subgroup or sensitivity analyses. As an observational study, causality cannot be definitively established despite careful adjustment for confounders, and residual confounding by unmeasured variables (e.g., medication adherence, autonomic dysfunction, or microvascular disease burden) cannot be fully excluded.

A key limitation of this study was the lack of detailed data on important confounders that may have influenced SBPV, including antihypertensive treatment strategies (e.g., drug class, timing, and dosing), cardiac arrhythmias such as atrial fibrillation, and markers of autonomic dysfunction. The absence of these variables may have introduced residual confounding and limited the ability to fully account for factors affecting short-term BP dynamics. Another limitation related to heterogeneity in BP measurement. Invasive monitoring provided continuous, high-resolution hemodynamic data, whereas non-invasive methods were intermittent and may have underestimated rapid fluctuations in SBP. Because information on monitoring modality and measurement frequency was not available, stratified or sensitivity analyses could not be performed, which may have introduced measurement-related bias.

Moreover, the SBPV threshold identified through ROC analysis was both derived and evaluated within the same cohort, raising concerns regarding potential overfitting and optimistic estimation of predictive performance. In the absence of internal or external validation, this threshold should be considered exploratory and requires confirmation in independent cohorts. Notably, heterogeneity in BP monitoring is an important limitation. Invasive monitoring provides continuous, high-resolution data, whereas non-invasive methods are intermittent and may underestimate variability. As modality-specific data were unavailable, sensitivity analyses were not feasible. This may introduce measurement bias and should be considered when interpreting results.

A further limitation concerns estimation of cerebral autoregulation indices. Mx and COx are ideally derived from continuous high-resolution data, but were approximated here using intermittent measurements, potentially reducing accuracy. Inclusion of patients without invasive monitoring may add variability. Thus, findings should be interpreted cautiously, and validated in studies with continuous monitoring. Additional clinical factors, including mechanical ventilation, vasopressor use, and pre-admission antithrombotic therapy, may influence both BP variability and outcomes. These variables were not included in the primary multivariable models due to the limited sample size and risk of overfitting, and therefore residual confounding cannot be excluded.

Other limitations were that Renal function and acute kidney injury were not assessed in the present study. Given evidence from prior trials such as ATACH-2^74,75^ suggesting potential renal complications associated with aggressive BP management, the absence of renal outcome data represents a limitation. Future studies should incorporate systemic outcomes, including renal function, to better understand the broader physiological impact of BP variability in ICH. Additionally, BP was measured invasively only during the acute hospitalization period, precluding evaluation of long-term variability and its influence on chronic recovery or recurrent events. The study also did not account for treatment-related factors such as timing and intensity of antihypertensive therapy, which could influence both BP variability and outcomes. Another limitation is the lack of external validation or replication in independent cohorts, which would strengthen confidence in the identified SBPV thresholds. Finally, neuroimaging and autoregulation measurements were performed at discrete time points, potentially missing dynamic changes over time. Future multicenter studies with larger, more diverse populations, continuous multimodal monitoring, and interventional designs will be essential to confirm these findings and determine whether targeted modulation of SBPV can improve outcomes after ICH.

## Conclusion

This study demonstrates that high early SBPV is a strong, independent predictor of hematoma expansion, secondary brain injury, and poor functional recovery following spontaneous supratentorial ICH. The findings suggest that excessive short-term fluctuations in SBP may impair cerebral autoregulation, promote perihematomal edema formation, and ultimately worsen neurological outcomes.

These observations highlight the clinical relevance of dynamic BP monitoring in the acute phase of ICH. Traditional management has primarily focused on lowering mean SBP; however, our results indicate that stabilizing BP variability itself may be equally important for preventing secondary injury. These findings highlight the potential clinical relevance of systolic BP variability as a marker of hemodynamic instability associated with adverse outcomes in intracerebral hemorrhage. Rather than supporting a fixed threshold, the results suggest that increasing levels of BP variability are linked to progressively higher risk, underscoring the importance of careful and individualized BP management. Future research should aim to validate these findings in larger, multicenter cohorts and to determine whether therapeutic interventions specifically targeting SBPV reduction—such as tailored infusion protocols or advanced closed-loop BP control systems—can translate into improved radiological and functional outcomes.

## Data Availability

The datasets generated and/or analyzed during the current study are available from the corresponding author on reasonable request.

## Abbreviations

ICH: Intracerebral hemorrhage
SBPV: Systolic blood pressure variability
BBB: Blood–brain barrier
BP: Blood pressure
SBP: Systolic blood pressure
Mx: Mean flow index
Cox: Cerebral oximetry index
GCS: Glasgow coma scale
